# Supplementary material for: Topology aware multitask cascaded U-Net for cerebrovascular segmentation
Source: PLoS One. 2024 Dec 5;19(12):e0311439. doi: 10.1371/journal.pone.0311439 (PMC11620396; doi:10.1371/journal.pone.0311439)
Supplement: Attachment — Submitted filename: response_to_reviewers.pdf [file pone.0311439.s001.pdf]

Rebuttal Letter

We would like to warmly thank the Editor for the management of the review process of this manuscript. We also thank the Reviewers for having taken the time to carefully study our manuscript and for their valuable comments and suggestions that induced improvements of the manuscript. A point-by-point response to each of the issues raised by the Reviewers is given in this document.

Reviewer #1

Reviewer:

This research paper addresses the critical topic of automatic 3D segmentation of the cerebrovascular network. It employs a U-Net model trained using Dice and centerline Dice losses, resulting in reduced training time while better preserving the network's topology compared to other known methods for cerebrovascular segmentation. While the topic is very important and the approach the authors use is innovative, the manuscript suffers from a critical deficiency that needs correction.

Major correction

#1 Questioning the quality of the ground truth, which normally used as a gold standard. In line 294, it is stated that the value of is expected to be B1 = 1 and the value of B0 = 1. The authors continue with the explanation that the ground truths are noisy and topologically incorrect, so the actual numbers they measured and presented in the results section are much higher (they are not even the same order of the magnitude).

How do the authors evaluate what is correct if the gold standard test is not correct? That puts into question all the conclusions in the paper and, therefore, this section needs to be re-written.

Authors:

We thank the two Reviewers for pointing out this issue and providing us with the opportunity to clarify. We acknowledge that the "ground truths" we employed are indeed noisy and topologically incorrect. To address this, we have replaced the term "ground truth" with "manual annotation" in the manuscript and provided a more detailed explanation as follows: "Unlike other metrics, Betti numbers characterize the topology of a structure independently of its proposed annotation. Analyzing the Betti numbers of a segmentation results then

requires comparing them with the true (ground truth) Betti numbers. From an anatomical point of view, the topology of the brain arterial network is well established. All arteries are connected, thus β_0 is equal to 1; there is one tunnel (the circle of Willis), thus β_1 is equal to 1; and no cavities are present, thus β_2 is equal to 0. However, the Betti numbers of the Bullitt and IXI annotations were computed, and much greater values for β_0 and β_1 were observed (see Table 1). This discrepancy is a typical issue with segmentation annotations of geometrically complex 3D structures. Manual annotation is a labor-intensive task, usually performed on 2D slices, making it difficult to accurately capture the 3D geometry and topology of the annotated structure. Moreover, the annotation guidelines rarely focus on topology, as voxel-wise metrics are the gold standard for segmentation evaluation. This leads to annotations that are suitable for voxel-wise analysis but often inadequate for topological analysis. Our goal is to obtain vascular segmentation that reflects the true topology of the brain arterial network. In the following, the ground-truth Betti numbers of the brain artery network ($\beta_0 = 1$, $\beta_1 = 1$, and $\beta_2 = 0$) are used as the reference for the segmentation Betti numbers."

Reviewer:

Related to this, what is the unit of measurement or scale on the Y-axis in Figures 7 and 8 ?

Authors:

The Dice and ClDice are dimension-less and then without unit. They are also normalized metrics (between 0 and 1). The Betti numbers are the ranks of the successive homology groups. They are also given without unit, whereas they represent the numbers of connected components, tunnels and cavities, respectively. In the initial version of the manuscript, HD_{95} and $ASSD$ (which are distance measures) were initially expressed in voxels. We consider that it would be more appropriate to express them in millimeters due to the non-isotropic nature of the images (voxel dimensions: $0.5 \times 0.5 \times 0.8$ mm^3). As a consequence, we have recomputed the HD_{95} and $ASSD$ in millimeters and modified the manuscript accordingly. This adjustment does not induce a significative change in the results nor alter the conclusions stated in the manuscript.

Reviewer:

Minor suggestions

The presented work needs some additional information regarding the data sets used to test this newly developed method for cerebrovascular segmentation. Including the additional information would underscore the strengths and the true value of this research in real-world clinical applications.

#1 Inter/Expert Segmentation Variability.

The ground truth segmentations were performed by experts. Is there any information from previously published data on the expert-to-expert (human-to-human) variability of segmentations? Specifically, are the observed differences between expert-to-expert segmentation results comparable to the differences measured in this study (newly described automatic method vs. other state-of-the-art automatic methods, i.e. automatic-to-automatic method comparison)?

Authors:

We thank the reviewer for highlighting a major difficulty in evaluating cerebrovascular segmentation methods. The annotation of blood vessels in 3D images is a very tedious and error-prone task. Even with semi-automatic tools, an expert takes several hours to annotate an image. This explains why there are very few publicly available datasets, particularly for cerebral vascular segmentation, which involves a very complex network. To the best of our knowledge, no publicly available cerebrovascular dataset provides annotations from more than one expert per volume. Consequently, it is difficult to evaluate the expert-to-expert variability quantitatively. We agree that this question is central and we are currently working on addressing it. However, we believe that it is out of the scope of the current article. The reviewer may look at the following preprint where we discuss the origin of this expert-to-expert variability and propose guidelines to reduce it: `https://arxiv.org/abs/2404.01765`.

Reviewer:

#2 Data Characteristics.

Was the method tested on a dataset from healthy patients, or was cerebrovascular pathology present? Pathological conditions such as leaky vessels, inflammatory cell infiltrates, and bleeding could interfere with segmentation and evaluation, potentially leading to decreased accuracy and increased training time. Although all methods compared in this study used the same datasets, and therefore the observed differences in the results are not caused by differences in the health status of the cerebrovascular network, mentioning this fact is important. It demonstrates that the newly described method can be utilized in various situations normally encountered in real life, and not only under certain conditions (for example, only in healthy young people, because that would defeat the purpose of developing this method in the first place).

Authors:

We thank the reviewer for raising a valid concern regarding our study. Both datasets we used consist solely of healthy subjects. We agree that it would be very interesting to compare the methods on patients with vascular diseases. However, to the best of our knowledge, there are no publicly available datasets with segmentation annotations for pathological patients that would allow us to conduct such an analysis.

We are currently working on an annotation protocol for the segmentation of brain arteries on a dataset of patients with ischemic stroke. In the meantime, we believe that working on healthy subjects is relevant because most of the brain arteries in pathological patients (e.g., patients with stenosis or aneurysms) are similar to those in healthy patients. The question of vascular connectivity remains crucial for patients with cardiovascular pathologies, although additional challenges will arise. We have added the following sentence to the future work discussion of the conclusion of the article: "Additionally, it would be valuable to compare the methods on datasets of patients with cardiovascular pathologies to evaluate the robustness and generalizability of the proposed approach."

Reviewer:

Technical comments.

#1 Organization of the Manuscript. *The results section should contain only the results, referencing appropriate tables and figures after each section or statement. Comparisons to other works and conclusions should be moved to the discussion section.*

Authors:

We thank the reviewer for this comment. We have reorganized the manuscript to make the separation between the results and discussion sections clearer.

Reviewer:

#2 Consistency in the Paper Organization and Presentation.

The methods section explains B0, B1, and B2 numbers, while the results figures contain B1 and B0 numbers. However, the authors continue to discuss only B0. The reason for this needs better clarification and consistency.

Authors:

We thank the two reviewers for their insightful comments on that point. In 3D, the third Betti number β_2 represents the number of cavities in the studied structure. Anatomically, a vascular network (and especially the flowing blood that is visualized in angiographic images) does not contain cavities in healthy subjects and in absence of acquisition artifacts, so β_2 should be equal to 0. In practice, we computed the β_2 scores for all segmentation results and we consistently obtained $\beta_2 = 0$ across all cases.

Given that β_2 did not provide additional useful information and was uniformly zero, we decided to remove this column from the result tables. We have also clarified this point in the metrics section of the manuscript: "Additionally, the value of β_2 was computed for both Bullitt and IXI datasets, it was observed

that the values were null for both manuals annotations and predicted segmentations (corresponding to the theoretical value). Therefore, the β_2 values are not presented or discussed in the following sections."

In the following, we complete our answer to explain the lack of analysis of β_1 results.

In the literature, research focused on improving the topology of vascular segmentations typically emphasizes enhancing connectivity (β_0). This emphasis is due to β_0 being the most crucial and interpretable parameter for subsequent clinical and research applications. Conversely, tunnels (β_1) are much harder to visualize and interpret and methods usually do not attempt to reduce them directly in segmentations. Additionally, they are more prevalent in the annotations, which we believe is primarily due to annotation noise rather than genuine topological errors. For these reasons, it did not appear relevant to analyze the β_1 results. However, we have decided to include them in our study to maintain transparency and provide a complete picture of our findings.

Reviewer:

#3 Abbreviations. Add a list of abbreviations (e.g., MRA, CTA, TOF-MRA) or define the meaning of each abbreviation in parentheses the first time it is mentioned in the text.

Authors:

We have carefully reviewed the manuscript to ensure that each abbreviation is clearly defined upon its first mention.

Reviewer #2

Reviewer:

This article examines the topic of automatic 3D cerebrovascular network segmentation using a cascaded U-net model, incorporating a novel method for evaluating segmentation loss with Dice losses. Researchers trained and evaluated their U-Net model in comparison with other leading skeletonization models, with a controlled experimental setting. The authors report improved accuracy and faster prediction times. In addition to its primary contribution, this research also offers additional value by proposing a more effective computation of Dice loss and providing an open code repository for further research access.

The explanation of the proposed U-net model is sound and very well documented.

The research's purpose, methods, and experiments are well articulated, but the descriptions of the dataset (subsection 4.1) and metrics (subsection 4.2) have drawbacks that raise concerns on validity of achieved results.

#1 *Based on statements on Betti number values for ground truth data "This shows that the ground-truth are noisy and not topologically correct" - This raises*

a significant challenge in evaluating segmentation methods; doesn't this directly affect the withdrawn conclusions of the study and raises questions on model's performance?

Authors:

See answer to Reviewer 1 regarding the same question.

Reviewer:

#2 *Regarding the omission of β_2 results due to no apparent cavities, could you clarify how this was determined? Was the absence of cavities verified visually, mathematically, or through a combination of both methods?*

Authors:

We thank the reviewer for allowing us to clarify this point. We have checked the absence of cavities in the segmentation results computationally. We have computed the β_2 scores for each segmentation result from each compared approach and obtained consistently a value equal to 0. We believe that it is not possible to evaluate the absence of cavities on a segmentation results because of the number and spreading of the vessels in the image and the difficulty of visualizing a 3D structure on a 2D screen. We have clarified this point in the metric section: "Additionally, the value of β_2 was computed for both Bullitt and IXI datasets, it was observed that the values were null for both manuals annotations and predicted segmentations (corresponding to the theoretical value). Therefore, the β_2 values are not presented or discussed in the following sections"

Reviewer:

#3 The dataset description in subsection 4.1 is rather short. The study cited for the publicly available dataset does not contain a reference for the dataset itself; it would be beneficial to include a direct link to the repository containing the dataset along with the citation. It is unclear whether the vessel segmentation was performed by one or more experts. If done by multiple experts, what about consistency of the segmentations (e.g., expert to expert comparison)?

Authors:

We thank the reviewer for this insightful comment. For this study, we used two publicly available datasets: Bullitt[1] [3] and IXI[2]. We have provided the links to the web page describing each dataset in the Datasets section of the manuscript. We have also clarified that the annotation comes from a unique expert for each volume. Unfortunately, as explained in our answers to Reviewer #1, there is currently no publicly available dataset with annotations from several experts.

[1] `https://public.kitware.com/Wiki/TubeTK/Data`
[2] `https://brain-development.org/ixi-dataset/`

This limitation prevents us from analyzing our results with respect to inter-expert variability.

Reviewer:

Subsection 4.1 notes that the skeletonize method from scikit-image was used for generating the skeleton ground truth, but does not discuss why this method was chosen over other existing methods (such as graph based etc.).

Authors:

We chose the Lee skeletonization algorithm [2] because it is the gold standard used in the vascular segmentation community [4, 5]. It is known to be an algorithm that provides accurate skeletons results. We have clarified this point in the manuscript in the Datasets section.

Reviewer:

Additionally, the written language of the article can be enhanced by using an objective and formal tone, replacing first-person pronouns (we, ours) with passive voice constructions.

Authors:

We appreciate the reviewer's suggestion for improving the language of the manuscript. In response, we have revised the manuscript to adopt a more objective and formal tone by replacing first-person pronouns with passive voice constructions where appropriate.

References

[1] Rougé P, Conze PH, Passat N, Merveille O. Guidelines for Cerebrovascular Segmentation: Managing Imperfect Annotations in the context of Semi-Supervised Learning. arXiv preprint arXiv:240401765. 2024;.

[2] Lee, T., Kashyap, R. & Chu, C. Building skeleton models via 3-D medial surface axis thinning algorithms. *CVGIP: Graphical Models And Image Processing.* **56**, 462-478 (1994)

[3] Aylward SR, Bullitt E. Initialization, noise, singularities, and scale in height ridge traversal for tubular object centerline extraction. IEEE Transactions on Medical Imaging. 2002;21(2):61–75.

[4] Todorov, M., Paetzold, J., Schoppe, O., Tetteh, G., Shit, S., Efremov, V., Todorov-Völgyi, K., Düring, M., Dichgans, M., Piraud, M. & Others Machine learning analysis of whole mouse brain vasculature. *Nature Methods.* **17**, 442-449 (2020)

[5] Nader, R., Bourcier, R. & Autrusseau, F. Using deep learning for an automatic detection and classification of the vascular bifurcations along the Circle of Willis. *Medical Image Analysis*. **89** pp. 102919 (2023)
